# A clinical pilot study on overdentures retained by two unsplinted narrow-diameter implants– oral health-related quality of life and masticatory function

**DOI:** 10.1186/s40729-025-00637-3

**Published:** 2025-07-16

**Authors:** Jaana-Sophia Kern, Esra Salin, Gregor Slavicek, Florian Slavicek, Dirk Elvers, Frank Hölzle, Stefan Wolfart

**Affiliations:** 1 Department of Prosthodontics and Biomaterials, Center for Implantology, Uniklinik RWTH Aachen, Pauwelsstr. 30, 52074 Aachen, Germany; 2 Orehab Minds GmbH, Zettachring 2, 70567 Stuttgart, Germany; 3 Department of Oral and Maxillofacial Surgery, Uniklinik RWTH Aachen, Pauwelsstr. 30, 52074 Aachen, Germany

**Keywords:** Dental implants, Narrow-diameter, Implant prosthesis, Edentulous jaw, Implant-supported overdenture, Oral-health related quality of life, Chewing, Masticatory function

## Abstract

**Purpose:**

This prospective clinical pilot study aimed to evaluate oral health-related quality of life (OHRQoL) and both objective and subjective masticatory function following treatment with implant-supported overdentures on two reduced-diameter titanium-zirconium implants in the edentulous maxilla and mandible.

**Methods:**

Ten edentulous patients received two implants each in the maxillary and mandibular canine regions. Implant-supported overdentures were placed after a conventional healing period. Patients completed a shortened form of the Oral Health Impact Profile (OHIP-G14) and rated their chewing ability on a visual analog scale (VAS) at baseline and after 6, 12, 24, and 36 months. A standardized masticatory function test assessed objective chewing function. Statistical analysis comprised descriptive analysis and changes or differences in means were analyzed using different tests (Wilcoxon signed rank test, Friedman test).

**Results:**

Significant increases in subjective ability to eat various foods were noted after six months (*P* ≤.05). The mean total OHIP score decreased significantly from 33.2 at baseline to 4.7 at the 36-month follow-up. Objective masticatory efficacy showed significant improvement immediately after treatment (*P* =.005), with effects consistent over 36 months. At that time, six maxillary implants had been lost, while no mandibular implants had failed.

**Conclusions:**

In this clinical pilot study, the rehabilitation of a completely edentulous maxilla and mandible with two implants and a removable overdenture significantly enhanced OHRQoL and both subjective and objective masticatory ability over an observation period of 36 months.

## Introduction

Prosthetic restorations aim to restore function and aesthetics, with patient satisfaction being a key priority. Patient-reported outcomes in dentistry (dPROs) are commonly assessed using the Oral Health Impact Profile (OHIP), initially developed with 49 questions [1] and nowadays available in various shorter forms. The recent ITI Consensus Statement [2] on the impact of implant-supported prostheses on patient-reported outcomes in edentulous patients, compiled validated Dental Patient-Reported Outcome Measures (dPROMS) for evaluating dPROS. This statement is based in part on a systematic review by Abou-Ayash et al. [3] and confirms that implant-supported prostheses improve dPROs in edentulous patients. Systematic reviews by Thomason et al. [4] and Andreiotelli et al. [5] already highlighted these benefits in 2007 and 2010.

Implant treatment for edentulous jaws is well-established, with studies demonstrating long-term success of implants and superstructures. A 2016 meta-analysis found low post-loading implant loss rates in both jaws, regardless of whether the prosthesis is removable or fixed [6].

However, implant survival appears correlated with implant number. Specifically, using fewer than four implants has not yet been recommended for the edentulous maxilla, as an estimated 5-year implant survival rate of only 69.7% must be anticipated [6]. The estimated 5-survival rate increases to almost 90% when the number of implants is increased to four [6]. A recent RCT confirmed this observation, showing implant survival rates of 89.5% for stud attachments and 96.3% for bars in the edentulous maxilla [5]. Notably, implant loss seems to be significantly higher with solitary attachments than with implants splinted by bars [7–10].

However, despite the unsatisfactory implant outcomes with a minimal number of only two implants in the maxilla, there is still insufficient evidence regarding whether this concept, which has long been established as the standard in the mandible, leads to an improvement in OHRQoL as well as masticatory function. Currently, the only evidence available is from Zembic et al. [11] who have demonstrated that compared to a conventional denture, the incorporation of an overdenture supported by two implants in the edentulous maxilla leads to a notable enhancement in several OHIP categories. Mo et al. [12] and Fonteyne et al. [13] observed a significant improvement in OHRQoL after the insertion of overdentures on three and four implants, respectively, in the edentulous maxilla. And also the immediate loading and fixed restoration of six implants in the edentulous maxilla seems to result in satisfactory patient-reported outcomes [14].

However, research has largely focused on the mandible. Studies on the mandible consistently show improved OHRQoL with implant-supported prostheses [3, 15–17]. Even a single central implant significantly enhances OHIP scores [18, 19] with additional benefits from a second or a third implant [3, 20]. Similarly, studies on masticatory function primarily investigate mandibular overdentures [19–22], showing general improvements but inconsistent degrees of enhancement. None of these studies included maxillary edentulous patients treated with two implants and an overdenture, which distinguishes our study from the previously published ones.

The primary objective of this prospective clinical pilot study was to evaluate implant survival and marginal bone loss of two narrow-diameter titanium-zirconium implants supporting complete overdentures in the maxilla and mandible over 36 months [23]. This publication presents secondary outcome data, including OHIP-G14 [24] scores, subjective masticatory ability, and standardized masticatory function test results.

## Methods

This clinical pilot study received approval from the Ethics Committee of the Medical Faculty at RWTH Aachen University (approval number: EK 167/13) and was carried out in the Department of Oral and Maxillofacial Surgery and the Department of Prosthodontics and Biomaterials, Center for Implantology, Uniklinik RWTH Aachen, Germany. The study was registered as a clinical trial on ClinicalTrial.gov on December 18, 2018, identifier NCT03777748. Patients were recruited consecutively between June 2014 and January 2016 and were included if they were completely edentulous and dissatisfied with their complete dentures. They had to have a class IV bone atrophy [25], a good oral hygiene, and a good general health (class I/II ASA classification). A maximum of 10 cigarettes per day was allowed. Patients had to have given written informed consent prior to inclusion in the study.

The following section provides a brief overview of the surgical and prosthetic procedures. For a more comprehensive description, please refer to the publication by Kern et al. [14]. In a two-stage approach, patients received two reduced-diameter titanium-zirconium implants (Roxolid, RN SLActive Standard implants, 3.3/10 mm; Straumann) in the maxillary and mandibular canine regions. After 5 to 6 months the definitive overdentures were incorporated. They were attached to stud-attachments (either CM LOC or CM LOC flex attachments with possible divergence correction from 40 to 60°; both Cendres + Métaux). All overdentures had a cobalt chromium framework, and the maxillary overdentures were fabricated without palatal coverage.

Four weeks after prosthesis placement patients were examined for the first time (baseline). After that regular follow-ups were performed at 6, 12, 24, and 36 months. In addition to the dental examination, probing depth, bleeding and plaque index, standardized periapical radiographs were taken at baseline, and after 12 and 36 months to evaluate marginal bone levels around the implants. Technical and biological complications were recorded.

Patients filled in the OHIP-G14 questionnaire at every appointment, which assesses specific aspects related to psychological, physical, and social limitations, as well as discomfort and pain associated with the implant-supported overdentures. Responses were recorded using a 5-point Likert scale, ranging from 0="never" to 4="very often". Higher scores indicate a lower quality of life related to oral health. The highest and therefore most negative value is 56. Subjective masticatory function was evaluated using a VAS, which addressed various food types with differing consistencies (from soft to hard, e.g., bread, meat, carrots). A score of 0 (at the far left of the scale) indicated “unable to chew at all”, while a score of 10 (at the far right) indicated “able to chew without difficulty.”

**Fig. 1:**
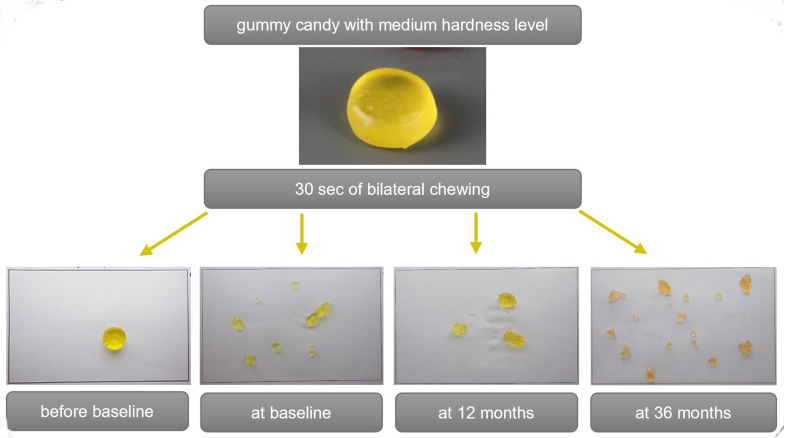
Example of standardized chewing function test of one patient. The top image shows the unchewed sample, while the four lower images depict the chewing sample after 30 s of chewing on both sides before baseline (with conventional dentures in both jaws), at baseline, 12, and 36 months

To assess objective masticatory function, a standardized masticatory function test according to Slavicek et al. [26, 27] was employed, utilizing standardized chewing samples based on gummy candies in three different hardness levels (soft/green, medium/yellow, hard/red). A complete masticatory function test consists of a total of 9 chewing sequences which were performed by every patient (right-sided, left-sided, and bilateral chewing for 30 s, each assessed for soft, medium, and hard consistencies). The chewed test food was collected in a sieve and the patients rinsed their mouths thoroughly with water. The rinsing fluids were added to the sieve to recover all the test food particles. The particles were then separated on a special sheet. Figure 1 shows the sheets of one patient who performed her chewing test at different time points. The sheets were photographed using a specialized stand equipped with a mounted camera and subsequently analyzed digitally. The number of chewed particles was counted. The geometric dimensions and volume of the particles were measured to determine the size distribution. The chewing test was performed prior to implant therapy (before baseline), at baseline, and at 6, 12, 24, and 36 months.

Descriptive analysis (mean, median, standard deviation, frequencies) was performed for OHIP total scores and individual questions. The Wilcoxon signed-rank test assessed significant changes over time. Subjective masticatory function (VAS) was averaged per patient, and chewed particles were compared to standard values. Chewing ability changes were analyzed using the Friedman test. Spearman’s rank correlation examined relationships between OHIP and masticatory function. Implant loss effects were assessed. Missing data were handled as user-defined missings in SPSS. Significance was set at *P* <.05. Effect sizes (Cohen’s d) enabled comparison with OHIP-14 literature. Analyses were conducted in IBM SPSS 29 and the freeware Psychometrica (www.psychometrica.de).

## Results

Ten patients were initially included in this clinical pilot study (baseline characteristics in Table 1). One patient died before the 12-month follow-up, leaving nine patients who completed the 36-month observation period. These nine patients completed the OHIP-G14 in full at all examination time points, as well as the VAS for subjective masticatory ability and the masticatory function test.

**Table 1:** Baseline characteristics

Variable	Value (*n* = 10)
Age, mean (range)	mean age 65.3 (58–79) years
Sex, n (%)	Male: 4 (40%), Female: 6 (60%)
ASA classification, n (%)	ASA II: 10 (100%)
Smoking status, n (%)	Current Smokers: 6 (60%) Never smokers: 4 (40%)
Jaw classification	Class IV (100%)
Previous denture experience	Yes: 10 (100%)
Pre-treatment OHIP total score, mean ±SD	33.22 ±13.85

Details on implant survival are summarized in Table 2, which was previously published in the paper by Kern et al. [14]. A total of six maxillary implants were lost over the 36-month follow-up period. The implant survival rate in the mandible was 100%. The first three implant losses in the maxilla occurred at 2, 6, and 11 months in two patients. By the 36-month follow-up, three additional implants had failed. In case of an implant loss, the overdenture was converted to a conventional complete denture with a closed palate. Moreover, 21 issues with retention inserts were recorded as early as six months post-treatment. By the final follow-up at 36 months, a total of 92 complete losses or retention failures of the inserts had occurred. Additionally, 34 cases of wear on the attachment or the titanium housing were documented.

**Table 2:** Details on implant losses (shortened version of table published in Kern et al. [23])

Patient	Region of implant loss	Sub-merged healing yes/no*	Highest probing depth in mm (at follow-up before loss)	BOP yes/no (at follow-up before loss)	mMBL in mm (mesial and distal; last x-ray before loss)	Time of post-loading implant loss (months)	Smoker yes/no	Type of attachment
1	013	no	3	no	1.7 2.1	29	no	CM LOC flex
5	013	yes	9	yes	1.9 1.4	16	yes	CM LOC flex
	023	yes	9	yes	0.9 1.0	11		CM LOC flex
8	023 (implant fracture	yes	6	yes	2.3 0.2	6	no	CM LOC flex
10	013	no	2	no	6.9 7.0	2	yes	CM LOC flex
	023	no	3	yes	3.4 0.2	36		CM LOC flex

*some implants were exposed spontaneously during the healing period

**Fig. 2:**
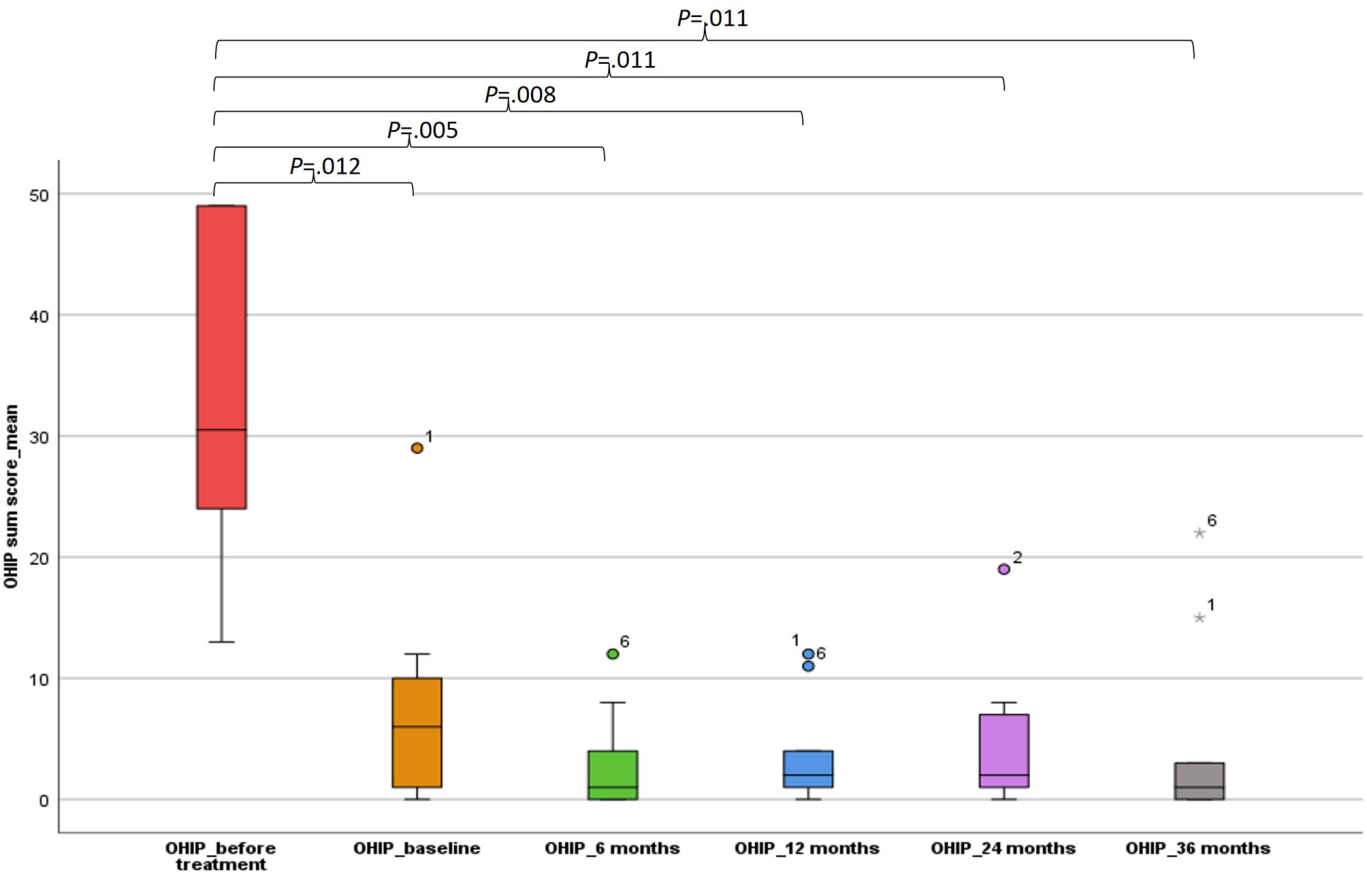
Box plot of OHIP-14 sum score before baseline, at baseline, and after 6, 12, 24, and 36 months

**Fig. 3:**
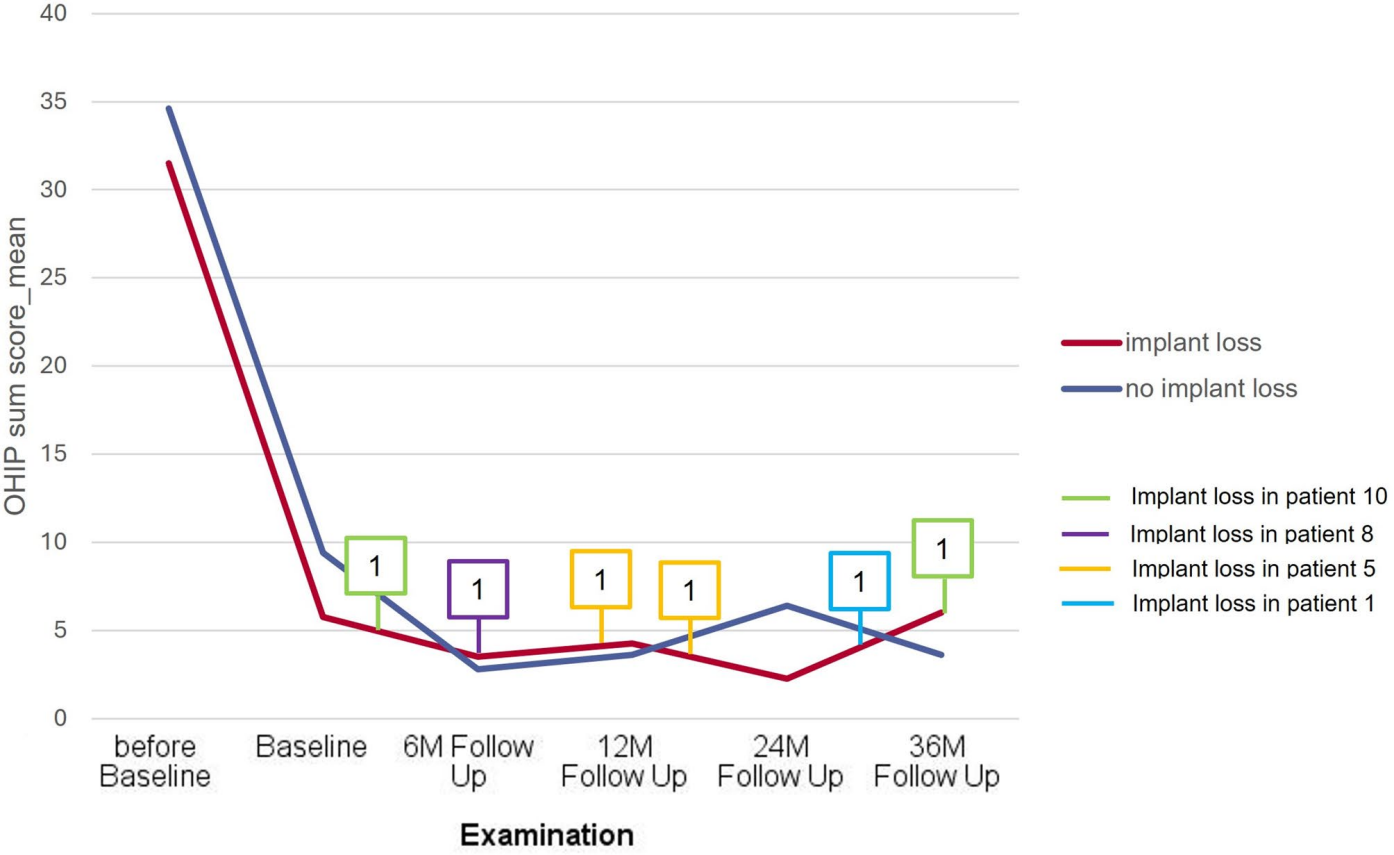
Comparison of OHIP-14 sum scores of patients with implant loss in the maxilla (*n* = 4) and patients without implant loss (*n* = 5) before baseline, at baseline, and after 6, 12, 24, and 36 months. Boxes indicate when which patient lost an implant. There was no significant difference between the groups with and without implant loss

The medians of the OHIP sum scores for all patients are presented as a box plot in Fig. 2. A significant improvement in OHIP scores was observed at all follow-up time points compared to pre-baseline values (Fig. 2). While the mean OHIP sum score for all patients prior to treatment was 33.22 ± 13.85 (Min 13–Max 49), which indicates a moderate to severe impairment of oral health-related quality of life, it had already decreased to an average of 7.6 ± 8.48 (Min 0–Max 29) by baseline, i.e., four weeks after the insertion of the overdentures. The best OHIP score of 2.8 ± 4.1 (Min 0–Max 12) was recorded at the six-month follow-up. At this time, two maxillary implants had already been lost in two patients (Fig. 3). Thereafter, a slight increase was observed, reaching a maximum value of 4.67 ± 8.1 (Min 0–Max 22) at 36 months. At this time, four more maxillary implants in two other patients had failed (Fig. 3). Implant loss did not have a significant effect on OHIP outcomes.

Looking at the individual categories of the OHIP survey, the two questions about tension and stress related to the teeth, mouth or dentures prior to treatment led to the highest reported values (36 and 33, respectively), indicating a moderate to severe impairment of the OHRQoL. Patients reported the least impairment of their OHRQoL when asked about feelings of insecurity regarding their teeth, mouth and dentures. Here, the total score before baseline was only 9 and decreased to 0 after 6 months already. The effect size (Cohen’s d) was calculated to compare the present pilot study with three reference studies from the literature that also utilized the OHIP-14. Table 3 provides an overview of the results.

**Table 3:** Comparison of OHIP sum score means with data from literature

Study	Treated jaw	Type of prosthesis	Number of implants per jaw	OHIP sum score (mean/SD) before baseline and after 1 year	OHIP sum score (mean/SD) before baseline and after 3 years	Effect size (Cohen’s d)
Present clinical pilot study	maxilla and mandible	overdenture	2	32.20 ±13.38 3.89 ±4.57	32.20 ±13.38 4.67 ±8.09	
Matthys et al. (2019)	mandible	overdenture	2	18.20 ±12.50 2.73 ±4.78		-0.378
Fonteyne et al. (2021)	maxilla	overdenture	4		21.45 ±12.19 6.13 ±4.91	-0.954
Sagheb et al. (2024)	mandible	overdenture	1–3	30.4 ±11.42 2.6 ±1.48 (3 implants)		-0.366

**Fig. 4:**
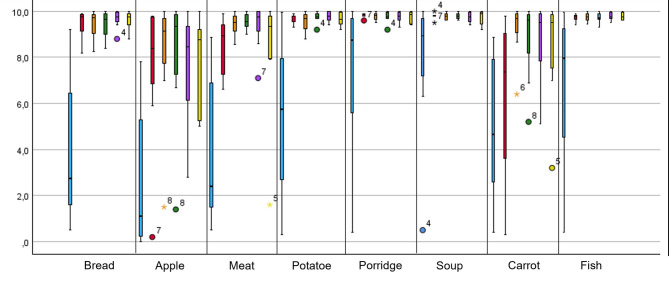
Box plot of visual analog scale of perceived masticatory ability for different types of food before baseline (blue), at baseline (red), after 6 months (orange), 12 months (green), 24 months (purple), and 36 months (yellow). There were significant differences between pre-treatment reports and reports at every post-treatment time-point except for soup and carrot. Exact *p*-values to be found in Table 4

When examining the results of subjective masticatory ability, significant improvements could be observed when comparing pre-baseline to baseline and to the time points at 6, 12, 24, and 36 months. While a majority of patients initially found it difficult to eat even relatively soft foods such as bread or potatoes before the insertion of the overdentures, a noticeable improvement was observed already four weeks after insertion, which persisted throughout the entire study period. For all assessed foods, the VAS rating indicated a significant improvement in subjective chewing ability except for soup after 36 months and carrot at baseline (Fig. 4; Table 4). A particularly notable improvement was observed in the ability to chew bread. The ability to chew hard foods such as carrots and apples was also rated significantly better compared to before the insertion of the overdenture, although, as expected, chewing ability for these foods was generally rated lower. Furthermore, when comparing the time point baseline to 6, 12, 24, and 36 months, patients continued to rate their chewing ability as improved.

**Table 4:** Exact *P*-values for VAS comparison of perceived masticatory ability for different types of food

Food item	Bread	Apple	Meat	Potatoe	Porridge	Soup	Carrot	Fish
***P*** **value**								
Pre vs.baseline Pre vs. 6 months Pre vs. 12 months Pre vs. 24 months Pre vs. 36 months	0.005 0.005 0.008 0.008 0.012	0.011 0.005 0.008 0.008 0.012	0.005 0.005 0.008 0.008 0.012	0.009 0.007 0.008 0.008 0.012	0.012 0.011 0.042 0.018 0.050	0.019 0.014 0.035 0.012 0.063	0.110 0.005 0.011 0.008 0.012	0.007 0.007 0.008 0.008 0.012

**Fig. 5:**
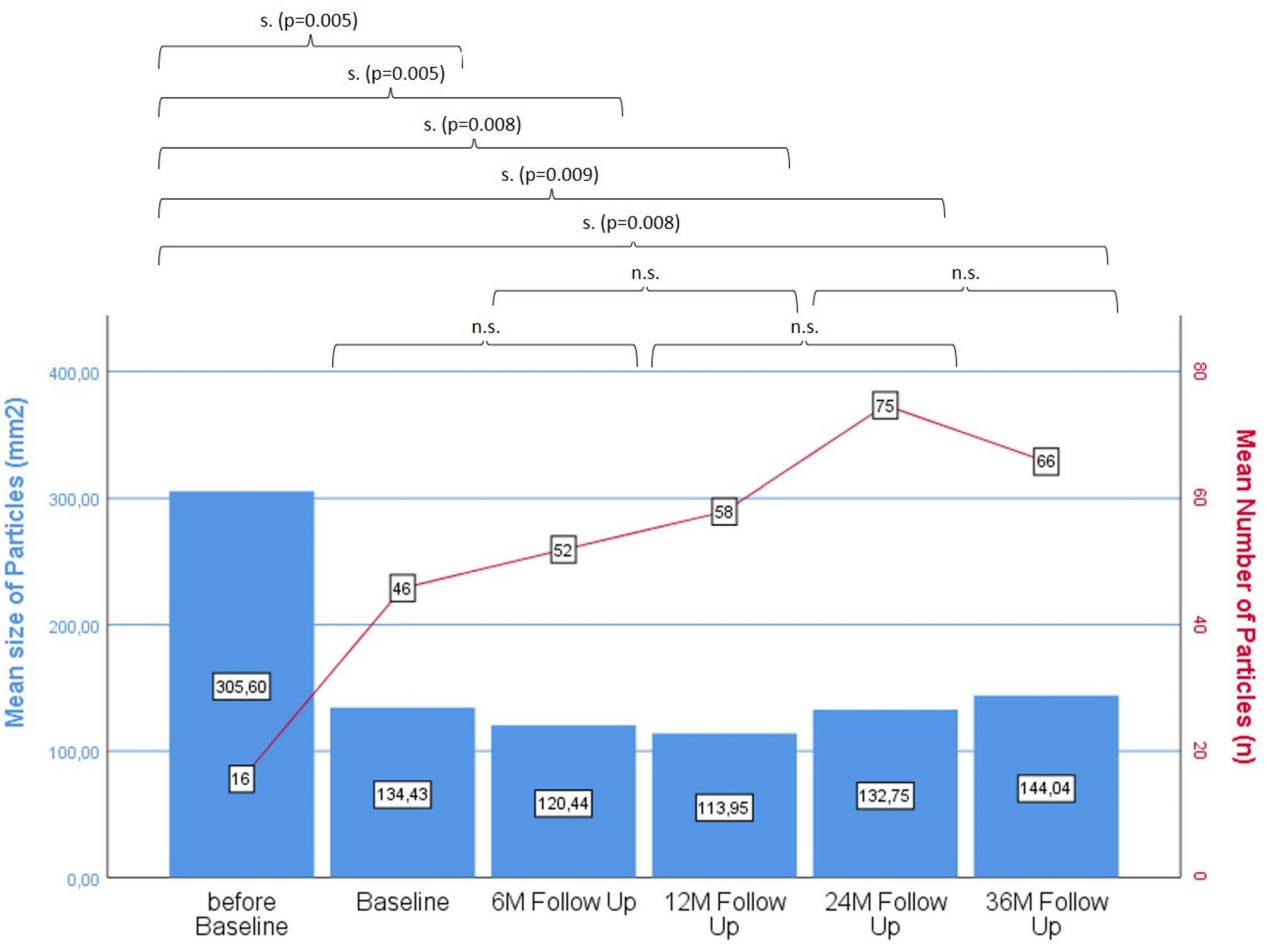
Results of masticatory function test: mean size (mm²) and mean number (n) of chewed particles before baseline, at baseline, and after 6, 12, 24, and 36 months

**Fig. 6:**
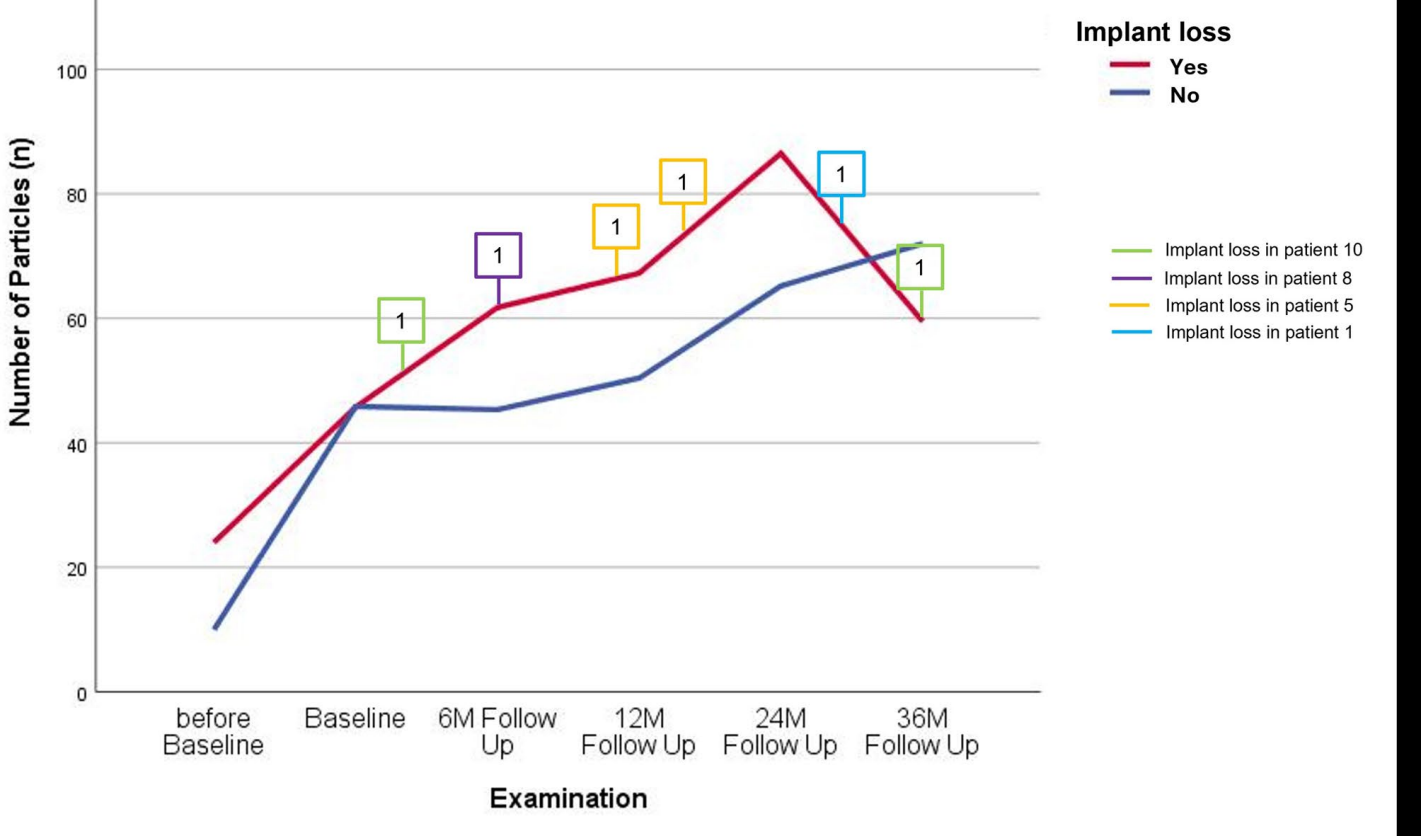
Comparison of mean number of chewed particles of patients with implant loss in the maxilla (*n* = 4) and patients without implant loss (*n* = 5) before baseline, at baseline, and after 6, 12, 24, and 36 months. Boxes indicate when which patient lost an implant. There was no significant difference between the groups with and without implant loss

This improvement in perceived masticatory ability correlated with the objective masticatory function that was assessed using the described standardized test method. The test demonstrated a significant improvement for all patients from baseline onward (Fig. 5). This was reflected in an increase in the average number of chewed particles (from *n* = 16 to *n* = 75 after 24 months) as well as a reduction in particle size from 305.6 mm² before baseline to a lowest value of 120.4 mm² at 6 months. These differences were significant at all time points compared to the before-baseline situation. However, with prolonged wearing time (after 36 months), it was observed that the mean number of chewed particles decreased again, and the average particle size slightly increased, indicating a slight deterioration in chewing ability. When comparing the number of chewed particles in patients who experienced implant loss with those who did not, a decrease in the number of particles in the implant loss group after 24 months was observed (Fig. 6). However, this difference was not significant.

## Discussion

This clinical pilot study evaluated two diameter-reduced titanium-zirconia implants with overdentures in edentulous jaws, assessing OHRQoL and masticatory function. The aim was to determine whether this minimal concept improved outcomes compared to conventional complete dentures and whether treating both jaws provided additional benefits over mandibular treatment alone.

Significant improvements were observed in OHIP scores, subjective masticatory function, and objective masticatory performance compared to the initial condition with conventional complete dentures. These findings align with previous clinical studies [12–14, 16] and systematic reviews [3, 17], and the ITI Consensus Statement [2], which confirm the benefits of implant-supported fixed or removable prostheses over conventional complete dentures. At 12 and 24 months, minor declines in OHIP scores and masticatory function were noted. Although implant loss did not seem to be the primary cause, frequent technical complications with anchorage elements or wear of the prosthetic teeth might have contributed. Implant survival in the maxilla was markedly low, with 6 out of 20 implants failing during the observation period. While complications with retention inserts were reported, their potential clinical consequences and possible association with implant loss were not fully explored. It is plausible that repeated technical issues, such as wear or diminished retention, may have contributed to mechanical overload or reduced prosthesis stability, thereby indirectly affecting implant survival. Future studies should investigate the nature, frequency, and consequences of prosthetic complications in greater detail, ideally incorporating long-term follow-up. The high failure rate raises concerns regarding the clinical applicability of the minimal two-implant concept in the edentulous maxilla, particularly when using diameter-reduced implants. In light of these findings, this approach should only be used with great caution and cannot currently be recommended for routine clinical application in the maxilla.

A comparison with Matthys et al. [16] (*n* = 90, mandibular overdentures with two implants and ball or stud attachments) yielded a small to moderate effect size (Cohen’s d=-0.378), suggesting that patients in the reference study reported slightly better OHIP-14 scores after intervention and that additional maxillary implants may not provide substantial improvements in OHIP scores. However, differences in sample sizes limit direct comparisons. Sagheb et al. [20] included ten edentulous patients and loaded three mandibular implants sequentially (first one, then two, then three), demonstrating that even a single implant led to improvements in OHIP scores, as also shown by Waltenberger et al. [18]. Post-treatment comparisons between the present study and Sagheb et al. [20] resulted in Cohen’s d=-0.366, indicating similar effectiveness, though variability seems to have been higher in the present study (SD: 8.09 vs. 1.48).

A comparison with Fonteyne et al. [13] (16 maxillary implant overdenture patients, four implants per jaw) showed a strong effect size (Cohen’s d=-0.954), with higher OHIP scores (6.14 vs. 4.67 in the present study). However, in Fonteyne et al. [13] only six of 21 patients had lower jaw dentures, while others had a natural mandibular dentition. Differences in baseline OHIP-14 values (33.22 vs. 21.45) must also be considered, as a higher initial impairment in this study may have allowed for a greater relative improvement. While effect sizes offer insights into patient-reported outcomes, their clinical significance remains uncertain due to methodological limitations. Differences in study design, patient selection, treatment protocols, and sample sizes likely influenced observed discrepancies, limiting generalizability.

The Slavicek masticatory function test is a validated method for assessing chewing efficiency, minimizing subjective bias [19, 20]. However, results may vary depending on patient characteristics. Available data on objective chewing efficiency with implant-supported restorations in the edentulous jaw remain limited, but clinical studies indicate improved masticatory function. Jasser et al. [22] investigated 20 patients with a chewing gum test and found significantly better masticatory efficiency in patients with mandibular fixed implant restorations compared to conventional complete dentures. Sagheb et al. [20], also using a chewing gum test, demonstrated significant improvements after the use of only one implant.

A key limitation of the present study is that the pre-treatment reference consisted of insufficient complete dentures. As all patients were dissatisfied with their dentures, their initial OHRQoL and masticatory function were relatively poor. Therefore, the observed improvements do not necessarily indicate that similar outcomes could not have been achieved with well-fitted conventional dentures– particularly in the upper jaw. A comparison group receiving conventional complete dentures or treatment with more than two implants per arch would have improved the clinical relevance of the study. Without such controls, it remains uncertain whether the observed benefits can be attributed specifically to the minimal implant concept or would also be achieved using more conventional or more extensive treatment protocols. Nonetheless, the study shows that two implants in both the maxilla and mandible can support favorable OHRQoL and masticatory function over a period of 36 months, even though nearly 50% of patients experienced implant loss in the maxilla, necessitating modified overdentures with palatal coverage. Importantly, this subgroup did not show a significant decline in OHRQoL or masticatory function.

Some studies show a positive effect of implant-supported dentures in the edentulous maxilla concerning dPROs [11, 13, 14] but there is a trend suggesting that maxillary implants play a lesser role than mandibular ones. This is supported by comparisons within this study and findings that edentulous patients generally adapt better to maxillary than mandibular dentures [15]. Individuals with a well-preserved alveolar ridge may not experience notable improvements in function or comfort with implant overdentures [4]. However, research on mandibular implant overdentures consistently demonstrates significantly higher patient satisfaction and a better OHRQoL compared to complete dentures [5].

The limitations of the present study have already been highlighted. As this was a clinical pilot study, the small sample size inherently limits the generalizability of the findings and increases the likelihood of variability in results. Accordingly, these results should be interpreted with appropriate caution. Future larger studies are necessary to validate these findings and further explore the impact of implant-supported prostheses on OHRQoL and masticatory function.

## Conclusions

Treatment with two implants and an overdenture in the maxilla and mandible significantly improved OHRQoL, perceived masticatory function, and objective masticatory performance compared to conventional complete dentures. However, this study does not provide evidence that additional maxillary implants yield significantly greater improvements in OHRQoL and masticatory function compared to mandibular treatment alone.

## Data Availability

The datasets generated and analyzed during the current study are not publicly available due to privacy reasons but are available from the corresponding author on reasonable request.
